# Significance of Serum Total Oxidant/Antioxidant Status in Patients with Colorectal Cancer

**DOI:** 10.1371/journal.pone.0170003

**Published:** 2017-01-19

**Authors:** Rong Wu, Jiafu Feng, Yuwei Yang, Chunmei Dai, Anyang Lu, Jie Li, Yao Liao, Miao Xiang, Qingmei Huang, Dong Wang, Xiao-Bo Du

**Affiliations:** 1 Department of Oncology, MianYang Central Hospital, Sichuan, People’s Republic of China; 2 Department of Surgery, Southwest Medical University, Sichuan, People’s Republic of China; 3 Department of Clinical Laboratory, MianYang Central Hospital, Sichuan, People’s Republic of China; 4 Emergency Department, MianYang Central Hospital, Sichuan, People’s Republic of China; 5 Department of Surgery, MianYang Central Hospital, Sichuan, People’s Republic of China; Kermanshah University of Medical Sciences, ISLAMIC REPUBLIC OF IRAN

## Abstract

Oxidative stress is involved in a variety of diseases. Prospective studies investigating the relationship between oxidative stress biomarkers and the status and development of colorectal cancer (CRC) are scarce; previous studies have failed to establish a relationship between the serum total oxidant/antioxidant status and CRC. Therefore, we compared the total serum oxidant/antioxidant levels of CRC patients and healthy subjects, and analyzed their clinical significance in the CRC. Fasting blood samples from 132 CRC patients and 64 healthy subjects were collected. Oxidative stress parameters, including total oxidant status (TOS) and total antioxidant status (TAS), were measured, and the oxidative stress index (OSI) was calculated. The TOS and OSI levels increased significantly (*P*<0.001) and the TAS level significantly decreased (*P*<0.001) in the CRC group compared to those in the healthy control group. Oxidative stress parameters differed significantly depending on the patient’s smoking and drinking status (*P*<0.05). The preoperative and postoperative levels of TOS, TAS, and OSI did not differ significantly between primary sites (colon/rectum) and clinical stages (*P*>0.05).However, the levels of TOS, TAS, and OSI were significantly different between patients with no metastasis and those with metastases to two organs (*P*<0.05) Finally, the parameters are affected by smoking and drinking, and subsequent research should be conducted excluding the relevant influencing factors.

## Introduction

Colorectal carcinoma (CRC) is one of the most common malignant tumors, and its morbidity and mortality rates have been increasing annually. The cancer statistics report for 2016 shows that CRC has the third highest incidence and mortality rates among all malignant tumors in developed countries such as the United States [1].In developing countries such as China, CRC also shows a high incidence rate, and its morbidity and mortality were the fifth highest among all malignant tumors in 2015[2].The age of disease onset was lower in China than in the United States; disease onset peaked at 60–74 years of age among Chinese patients, while it was 70 years among American patients [1–2].

Although the diagnosis and treatment of CRC have developed rapidly, its mortality rate is still high [3].This has a major impact on the patients as well as their family and community. Therefore, the occurrence and development of CRC have attracted the attention of many researchers, and an increasing number of studies have been conducted. Considering the current statistics, it is essential to understand the pathogenesis of the disease and the factors that might be involved in the process of disease development.

Oxidative stress occurs in response to the oxidative damage caused when the body’s antioxidative and scavenging activities cannot cope with the active oxidants produced by a harmful stimulant [4]. Oxidative stress involves macromolecular oxidative damage, induces tissue protein denaturation, DNA damage, and lipid peroxidation, and interferes with the body's normal metabolic activity, leading to the occurrence and/or development of diseases. It has been confirmed that oxidative stress is involved in a variety of diseases, such as pneumonia[5], pancreatitis[6], diabetic nephropathy[7], cardiovascular disease[8], nervous system disease[9], and cancer[10–13]. Reactive oxygen species (ROS) make up the majority of active oxides, and account for more than 95% of total oxides. ROS are involved in the occurrence and development of malignant tumors through the induction of DNA damage and genetic mutations, inhibition of apoptosis, and promotion of the proliferation, invasion, and metastasis of malignant cells [14]. For example, ROS can activate or increase the expression of matrix metalloproteinases, adhesion molecules, and epidermal growth factor and its receptor, and promote tumor cell metastasis in patients with CRC. Under the action of thymidine phosphorylase, ROS also stimulates tumor cell proliferation and invasion[15,16].

Previous studies have reported that oxidative stress is closely related to the occurrence and development of breast [10], thyroid [11], cervical [12], and prostate cancers [13]. However, in the past 20 years, prospective studies have investigated the relationship between biomarkers of oxidative stress and the occurrence and development of CRC [17]. In these studies, only one or several oxidants/antioxidants were measured separately, and studies reporting the relationship between serum total oxidant/antioxidant status and CRC are scarce. Although existing technologies allow for measurement of serum levels of each oxidant/antioxidant separately, they are time-consuming and labor-intensive, and might not be accurate for the following two reasons: (1) there might still be unknown oxidants/antioxidants in the serum; and (2) different types of oxidants/antioxidants in the same system could interact with each other and cause an additive or synergistic effect. Measuring only one or several individual oxidants/antioxidants in the serum is insufficient to establish a definitive relationship between oxidative stress and CRC.

Therefore, the total oxidant status (TOS) is usually used to estimate the overall oxidation state of the body[18]. Similarly, the total antioxidant status (TAS) is used to measure the overall antioxidant status of the body[19]. The oxidative stress index (OSI), which is the ratio of TOS to TAS [20], might be a more precise index of oxidative stress in the body because it is a comprehensive measurement of TAS and TOS. TOS, TAS, and OSI are oxidative stress parameters used to evaluate the overall oxidative stress status in the body. In the present study, we evaluated the levels of TOS, TAS, and OSI in healthy controls and CRC patients in order to analyze the relationship between the oxidative stress parameters and CRC, and to analyze the clinical significance of this index in CRC.

## Materials and Methods

### Subjects

From August 2014 to August 2015, 132 patients with CRC (76 men and 56 women; mean age, 58 ± 12.3 years; group A) and 64 healthy individuals(30 men and 34 women; mean age, 55 ± 12.5 years; group B) were enrolled in the present study. The basic characteristics of the study subjects are shown in Table 1. All patients were diagnosed with CRC based on histopathological biopsy evaluation. In group A, 106 of the patients were from the oncology department and had not previously undergone radiotherapy or chemotherapy, whereas 26 patients were from the surgery department and had undergone radical surgery; patients who had undergone resection of metastatic lesions were excluded. CRC patients who had not previously undergone radiotherapy or chemotherapy were stratified into groups: patients with the colon as the primary site were allocated to group C; those with the rectum as the primary site were allocated to group D; those with a history of smoking or drinking were allocated to group E; and those with no history of smoking and drinking were allocated to group F.

**Table 1:** Basic characteristics of subjects.

Characteristics	Group A (n = 132)	Group B (n = 64)	
**Age(years)**			*P* = 0.162
**Mean± S.D.**	58±12.3	55±12.5	
**Sex**			*P* = 0.159
**Male**	76	30	
**Female**	56	34	
**Smoking history**			*P* = 0.086
**Yes**	40	12	
**No**	92	52	
**Drinking history**			*P* = 0.170
**Yes**	39	13	
**No**	93	51	
**Histopathologic type**			
**Adenocarcinoma**	132		
**Others**	0		
**Primary lesion**			
**Colon**	67		
**Rectum**	65		
**Clinical stage**			
**Stage I**	6		
**Stage II**	25		
**Stage III**	34		
**Stage IV**	67		
**Histological grade**			
**High grade**	0		
**Moderate grade**	77		
**Low grade**	8		
**Middle-low grade**	10		
**Unknown**	37		

The present study was approved by the medical ethics committee of the MianYang Central Hospital, Sichuan Province, China (number: 2013042). All blood were obtained with the consent of the patients and healthy individuals, and written informed consent from the donors or the next of kin was obtained for use of the blood samples for research purposes.

### Blood samples

All subjects were asked to fast for 8–12 hours overnight. Approximately 4 mL of peripheral venous blood was drawn using a disposable venous blood collector (21-Ga×1″, BD) and collected in a blood collection tube (BD Vacutainer). Within 2 hours, blood samples were centrifuged at 3000 rpm for 15 minutes and serum samples were stored at -70°C until analysis, which was performed within 24 hours. Blood samples were collected from the patients who underwent radical surgery both preoperatively and 48 hours postoperatively.

### Measurement of TOS

TOS was measured using the Erel TOS method [18],which was based on the oxidation of ferrous ion to ferric ion in the presence of various oxidative species under acidic conditions. The level of ferric ion was measured using xylenol orange ona 7600–020 fully automatic biochemical analyzer (HITACHI). The test parameters were as follows: method, end-point measurement; temperature, 37°C; primary wavelength, 570 nm: secondary wavelength, 800 nm;R1 volume, 270 μL, R2 volume, 20 μL, and S, 35 μL; and reaction time, 5 minutes. The results were expressed in μmolH_2_O_2_ equivalent/L (μmolH_2_O_2_ equiv./L).

### Measurement of TAS

TAS was measured using the 2,2'-azino-di-3-ethylbenzthiazoline sulfonate (ABTS)^+^ colorimetric method. This assay depends on the ability of antioxidants in the serum to inhibit the formation of ABTS^+^ from the oxidation of ABTS by metmyoglobin (a peroxidase). The 5 mmol/L solution of Trolox (Couinaud dimethyl acrylate) was used as a standard for the calculation of antioxidant levels in the samples. The test parameters were as follows: method, end-point measurement; temperature, 37°C; R1 volume, 200 μL; R2 volume, 20 μL, and S, 5 μL. The reaction time was 10 minutes and the results were expressed in mmol Trolox equivalent/L (mmol Trolox equiv./L).

### OSI

OSI was calculated using the following formula: OSI (arbitrary units) = [(TOS, μmolH_2_O_2_equiv./L)/(TAS, μmol Trolox equiv./L)×100][21].

### Statistical analysis

In analysis of the basic characteristics of the subjects, ages were compared using the Student t-test. The sex of the patients, as well as their smoking and drinking history were compared using the Pearson chi-square test. The k-s test was performed to determine whether the data were normally distributed. If the data were normally distributed, the results were expressed as the mean ± standard deviation and/or as a range (minimum–maximum). The two groups were compared using the Student t-test and multiple sets of comparisons performed using ANOVA were presented as F values. If the data were skewed, the results were expressed as the median (P25–P75) and they were compared using the Kruskal–Wallis test. The results of the comparison were presented as chi-square (χ^2^) statistics. *P* values < 0.05 were considered statistically significant. All statistical analyses were performed using the SPSS statistical software v22.0 (IBM).

## Results

All subject demographics and clinical data are summarized in Table 1. There were no significant differences in age (*P* = 0.162), sex (*P* = 0.159), smoking history (*P* = 0.086), or drinking history (*P* = 0.170) between the patients and controls. The serum levels of oxidative stress parameters in CRC patients (group A) and healthy controls (group B) are shown in Table 2.

**Table 2:** Serum levels of oxidative stress parameters between CRC patients and healthy controls.

	Median(P_25_∼P_75_)	*P*
**TOS**		
**Healthy Controls**(**n = 64**)	14.20(11.80∼17.38)	
**Stage I**(**n = 6)**	16.85(10.48∼22.93)	0.419
**Stage II**(**n = 25)**	18.30(13.95∼28.25)	<0.001
**Stage III**(**n = 34**)	22.05(15.85∼27.80)	<0.001
**Stage IV(n = 67)**	20.40(16.80∼27.70)	<0.001
**TAS**		
**Healthy Controls**(**n = 64**)	1.77(1.69∼1.88)	
**Stage I**(**n = 6)**	1.60(1.39∼1.68)	0.006
**Stage II**(**n = 25)**	1.44(1.34∼1.60)	<0.001
**Stage III**(**n = 34**)	1.53(1.36∼1.64)	<0.001
**Stage IV(n = 67)**	1.54(1.40∼1.65)	<0.001
**OSI**		
**Healthy Controls**(**n = 64**)	0.80(0.64∼0.99)	
**Stage I**(**n = 6)**	1.05(0.74∼1.39)	0.136
**Stage II**(**n = 25)**	1.33(0.89∼2.04)	<0.001
**Stage III**(**n = 34**)	1.39(1.00∼2.10)	<0.001
**Stage IV(n = 67)**	1.34(1.04∼1.83)	<0.001

Compared to those in the controls, the serum levels of TOS as well as OSI were significantly increased (*P*≤0.001) in patients with CRC of stage II, III and IV, and TAS levels were significantly decreased in patients with CRC of stage I(*P*< 0.006), II (*P*< 0.001), III (*P*< 0.001), and IV (*P*< 0.001).

**Fig 1:**
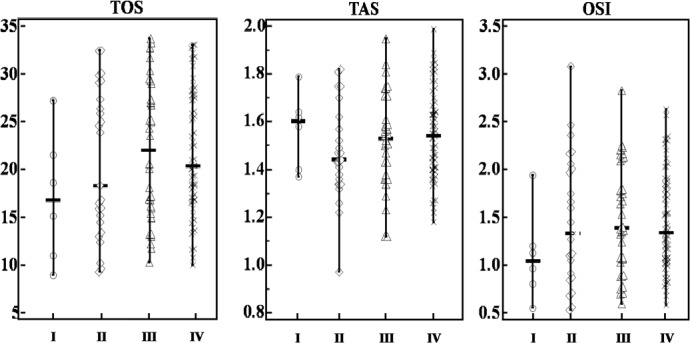
The distribution of serum TOS,TAS, and OSI levels in different clinical stages of CRC patients. “○,◇,▲,×”: Oxidative stress parameters of patients with stage I, II, III, and IV, respectively. “-” of vertical lines: median. Top of vertical lines: Maximum value. Bottom of vertical lines: minimum value.

Fig 1 shows the distribution of serum TOS, TAS, and OSI levels for different clinical stages of CRC. Multiple comparisons between subjects with different clinical stages of CRC showed that levels of TAS, TOS, and OSI did not differ significantly between different clinical stages (*P* > 0.05; Table 3).

**Table 3:** Serum levels of oxidative stress parameters in CRC clinical stages.

	CRC clinical stages(n = 132)	Median(P_25_∼P_75_)	Min.	Max.	χ^2^	*P*
**TOS**	**Stage I**(**n = 6**)	16.85(10.48∼22.93)	8.90	27.20	3.100	0.376
	**Stage II**(**n = 25**)	18.30(13.95∼28.25)	9.30	32.50		
	**Stage III**(**n = 34**)	22.05(15.85∼27.80)	10.30	33.70		
	**Stage IV(n = 67)**	20.40(16.80∼27.70)	10.00	33.10		
**TAS**	**Stage I**(**n = 6**)	1.60(1.39∼1.68)	1.37	1.79	3.695	0.296
	**Stage II(n = 25)**	1.44(1.34∼1.60)	0.97	1.82		
	**Stage III(n = 34)**	1.53(1.36∼1.64)	1.12	1.95		
	**Stage IV(n = 67)**	1.54(1.40∼1.65)	1.18	1.99		
**OSI**	**Stage I(n = 6)**	1.05(0.74∼1.39)	0.55	1.94	2.671	0.445
	**Stage II(n = 25)**	1.33(0.89∼2.04)	0.53	3.08		
	**Stage III(n = 34)**	1.39(1.00∼2.10)	0.59	2.83		
	**Stage IV(n = 67)**	1.34(1.04∼1.83)	0.58	2.63		

**Fig 2:**
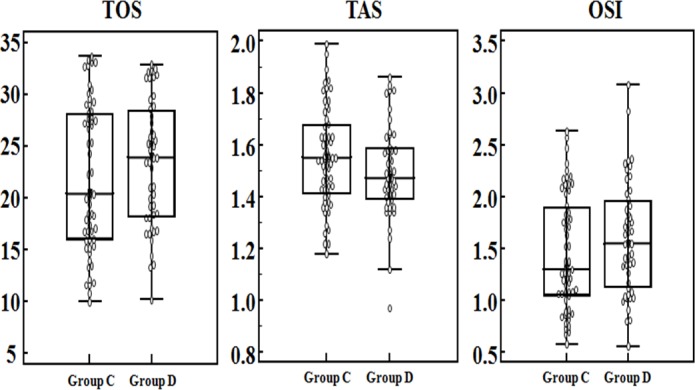
The distribution of oxidative stress parameter levels in different primary sites. 0: Serum TOS, TAS, and OSI levels of patients (group C: colon cancer patients; group D: rectal cancer patients).Box plot: inter-quartile range(from P_25_ to P_75_), “-”of Box plot: median.

**Fig 3:**
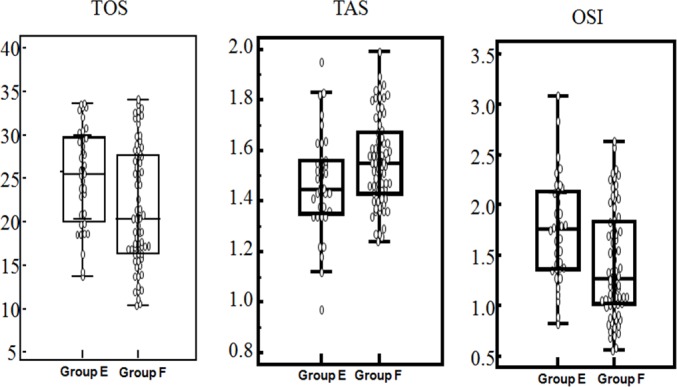
The distribution of oxidative stress parameter levels in CRC patients with smoking/drinking history. Serum TOS,TAS, and OSI levels of patients (group E: with smoking or drinking history; group F: patients with no smoking and drinking history). Box plot: inter-quartile range (from P_25_ to P_75_), “-”of box plot: median.

**Fig 4:**
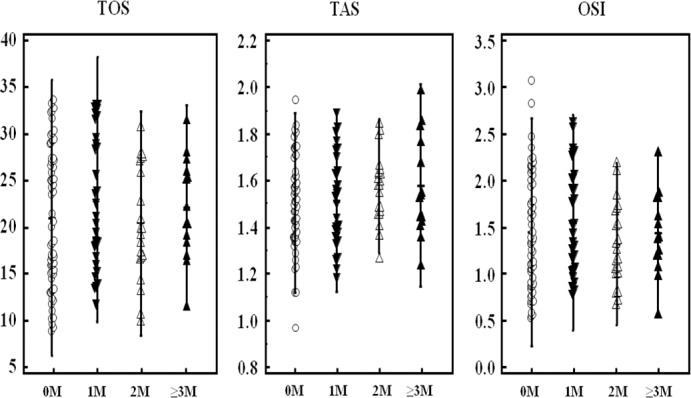
The distribution of oxidative stress parameters levels in CRC patients with different number metastatic site. “○,▼,△,▲”: Oxidative stress parameters of patients with no, 1, 2 and ≥3 metastatic sites, respectively. “OM”: no metastasis; “1M”: one metastatic site; “2M”: two metastatic sites, “≥3M”: ≥ three metastatic sites. “-”of vertical lines: mean, “|”: ±2SD.

Serum oxidative stress parameters in CRC patients who had not previously undergone radiotherapy or chemotherapy were used for patient stratification. Fig 2 shows the distribution of oxidative stress parameter levels in the CRC patients. The serum TOS, TAS, and OSI levels did not differ significantly between the primary sites (colon/rectum) (*P*>0.05; Table 4). Serum TOS (χ^2^ = 7.157, *P* = 0.007), TAS (χ^2^ = 4.909, *P* = 0.027), and OSI(χ^2^ = 9.460, *P* = 0.002) levels were correlated with the history of smoking/drinking in CRC patients (Fig 3, Table 4). In addition, the present study showed that oxidative stress parameters did not differ significantly between metastatic and non-metastatic CRC patients. However, a significant difference was observed between patients who had no metastasis and those who had two metastatic sites (Table 5). The relationship between tumor metastasis and oxidative stress parameters is demonstrated in Fig 4.

**Table 4:** Relationship between tumor primary lesions or smoking/drinking history and oxidative stress parameters.

	Groups(n)	Median(P_25_∼P_75_)	Min.	Max.	χ^2^	*P*
**TOS**	**C**(**59**)		10.01	33.72	0.898	0.343
	**D(47)**	23.90(18.10∼28.60)	10.22	32.93		
	**E(36)**	25.40 (19.65∼29.75)	13.33	33.31	7.157	0.007
	**F(70)**	20.00 (16.00∼27.35)	10.01	33.72		
**TAS**	**C(59)**	1.55(1.41∼1.68)	1.18	1.99	1.924	0.165
	**D(47)**	1.47(1.39∼1.59)	0.97	1.86		
	**E(36)**	1.45 (1.56∼1.35)	0.97	1.95	4.909	0.027
	**F(70)**	1.55 (1.43∼1.67)	1.24	1.99		
**OSI**	**C(59)**	1.30(1.05∼1.91)	0.58	2.63	2.048	0.152
	**D(47)**	1.55(1.12∼1.97)	0.56	3.08		
	**E(36)**	1.76 (1.36∼2.13)	0.82	3.00	9.460	0.002
	**F(70)**	1.27 (1.02∼1.84)	0.56	2.63		

Group C: patients with colon cancer; Group D: patients with rectal cancer; Group E: patients with smoking or drinking history; Group F: patients with no smoking and drinking history.

**Table 5:** Serum levels of oxidative stress parameters in no metastasis and metastatic patients.

	Mean± S.D.	*F*	*P*
**TOS**			
**No metastasis (n = 41)**	23.42±7.01		
**One metastatic site (n = 29)**	21.17±7.14	0.083	0.886
**Two metastatic sites (n = 22)**	20.45±6.02	2.254	0.043
**≥Three metastatic sites (n = 14)**	22.13±5.44	4.299	0.849
TAS			
**No metastasis (n = 41)**	1.49±0.19		
**One metastatic site (n = 29)**	1.52±0.19	0.616	0.558
**Two metastatic sites (n = 22)**	1.58±0.14	0.531	0.027
**≥Three metastatic sites (n = 14)**	1.58±0.22	0.221	0.493
TOS			
**No metastasis (n = 41)**	1.62±0.60		
**One metastatic site (n = 29)**	1.57±0.59	0.087	0.736
**Two metastatic sites (n = 22)**	1.32±0.43	3.752	0.014
**≥Three metastatic sites (n = 14)**	1.44±0.44	3.594	0.798

In a comparison of the preoperative and postoperative levels of serum oxidative stress parameters in 26 CRC patients who underwent radical surgery treatment, no significant differences were identified (*P*>0.05; Table 6).

**Table 6:** Serum levels of oxidative stress parameters in preoperative and postoperative patients.

	Status (n = 26)	Mean	S.D.	Min.	Max.	*F*	*P*
**TOS**	**Preoperative**	17.72	6.258	9.31	29.32	0.609	0.652
	**Postoperative**	19.13	7.044	7.12	28.53		
**TAS**	**Postoperative**	1.47	0.194	1.23	1.75	0.840	0.327
	**Postoperative**	1.62	0.375	1.08	2.23		
**OSI**	**Postoperative**	1.23	0.478	0.53	2.15	0.453	0.930
	**Postoperative**	1.25	0.582	0.46	2.19		

## Discussion

The incidence and mortality rates of CRC are very high and have been annually increasing worldwide. There have been recent advances in the research of tumor gene engineering technology and molecular biology. The diagnosis and treatment of CRC have significantly improved and treatment is no longer limited to surgery or chemotherapy and radiotherapy, since new treatment methods and drugs are available. Gene-targeted drugs and tumor immunetherapy has been shown to have a certain curative effect and good prospects for development. Despite these advances, the mortality rates for CRC have not decreased and it remains a direct threat to human health. Although many research studies have been conducted, the pathogenesis of CRC has not been fully explained.

A number of studies have confirmed that oxidative stress is associated with the pathogenesis of many diseases. In a study of malignant tumors, Feng et al. [10] measured the serum levels of OSI, TAS, and TOS in breast cancer, benign breast tumors, and healthy subjects. The serum oxidative stress parameters differed significantly between breast cancer patients and patients with benign breast tumors or healthy controls (*P*< 0.05). The serum levels of TOS and OSI were higher in patients with benign and malignant tumors than in healthy controls; however, TAS levels were significantly lower in patients with benign and malignant tumors than in healthy controls. Compared to the serum TOS level in patients with benign breast tumors, the serum TOS levels in patients with breast cancer were higher, while TAS levels were lower. The oxidative stress parameters differed significantly between cancer patients with different clinical stages. These findings suggested that the occurrence and development of breast cancer are closely related to oxidative stress. Wang et al. [11] showed that serum levels of TOS and OSI were significantly higher (*P*<0.001) in patients with thyroid cancer than in controls (*P*<0.001), and the serum levels of TAS were significantly lower in patients with thyroid cancer than in healthy controls. ROC curve analysis demonstrated that OSI was the best indicator for distinguishing patients with thyroid cancer from those with benign thyroid disease or healthy controls, followed by TOS and TAS. These results suggest that there is a relationship between oxidative stress and the development of thyroid cancer. Perše et al. suggested that different endogenous and exogenous factors may influence oxidative status and modulate the ability of gut epithelial cells to cope with damaging metabolic challenges [22]. However, the authors were not certain that oxidative stress is a consequence of the pathogenesis of CRC, and numerous studies showed that oxidative stress plays an important role in carcinogenesis [14,23,24]. This led us to investigate the levels of total oxidants/antioxidants in the sera of patients with CRC.

Blood samples were collected before radiotherapy and chemotherapy in the present study. Radiotherapy and chemotherapy can inhibit or kill cancer, which results in the production of a large number of free radicals, reduced antioxidant activity, increased oxidative stress, interferences with data measurement, and experimental errors. Santiago-Arteche et al.[25] also confirmed that total antioxidant capacity (TAC) was significantly lower in patients with CRC who received chemotherapy than in patients who did not receive chemotherapy. Therefore, in the present study we excluded the effects of radiotherapy and chemotherapy.

Our findings showed that oxidative stress is not related to the tumor primary site (colon/rectum), which is in turn related to the tumor load. However, there were no significant differences between the preoperative and postoperative levels of TOS, TAS, and OSI. Although surgery can remove a greater portion of the oxidative load of the tumor, surgical trauma can stimulate a stress reaction, which results in release of a large amount of free radicals, disruption of homeostasis of the oxidant/antioxidant system, and ultimately oxidative stress. In the present study, the majority of patients with CRC underwent open surgery followed by laparoscopy. Owing to severe open surgical trauma, oxygen exposure is obvious in vivo. Bukan et al. [26,27] suggested that open surgery could lead to oxidative stress, which would result in changes in oxidative stress parameters in vivo. Domestic scholars found that as a result of either open surgery or laparoscopic surgery, the postoperative plasma malondialdehyde levels of patients were increased, GSH-Px and antioxidant capacities were decreased, and oxidative stress persisted after open surgery[28]. In laparoscopic surgery, CO_2_ pneumoperitoneum is an independent factor of oxidative stress. Kontoulis et al. [29] established a CO_2_ pneumoperitoneum model in rats, and measured the malondialdehyde and peroxidase levels of the plasma and tissue. The results showed that their levels all increased with the induction of only CO_2_ pneumoperitoneum, and were positively correlated with surgical time. Tissue damage was also more severe under the microscope; therefore, it was concluded that pneumoperitoneum might lead to oxidative stress, and be positively associated with its oxidative stress. Luo et al.[30]confirmed these findings. In the present study, no significant differences in the oxidative stress parameters in CRC patients were observed between different clinical stages. One reason could be that the statistical analysis included patients who underwent postoperative adjuvant radiotherapy or chemotherapy. Our findings also showed there was a significant difference between the oxidative stress parameters of CRC patients who had no metastasis and those who had two metastatic sites. However, there were no significant differences between patients who showed no metastasis and those with either one or more than three metastatic sites. Stem from patients with three, one, or no metastatic sites included 36 patients who received postoperative adjuvant radiotherapy or chemotherapy, and was influenced by the surgical factors. In addition to the 36 patients with no metastases, the remaining cases showed local tumor invasion and were inoperable. The tumor burden in these cases was sometimes greater than that in patients with one metastatic site. Finally, the number of samples obtained from patients with more than three metastatic sites was too small, resulting in a large sampling size, low test efficiency, and misrepresentation of the true status. There were no significant differences in the serum levels of TOS and OSI between patients with stage I CRC and healthy individuals, which may be related to the small sample size.

The results of the present study suggested that the oxidative stress parameters in patients with CRC are affected by drinking and smoking. Tobacco and alcohol are currently recognized as risk factors for cancer. The International Cancer Research Institute had previously pointed out that tobacco is a leading carcinogen in many cancers, and CRC is no exception[31]. Approximately 10^15^ free radicals can be produced during the consumption of tobacco. Hydrogen peroxide, hydroxyl radicals, and superoxide anions can be produced by the reaction of the semiquinone radical in tar with oxygen in the air. The concentration of nitric oxide in tobacco is 500–1000 ppm, and it can react with superoxide anions to form a peroxide nitrite [32,33].Previous studies have shown a significant difference in the levels of oxidants and antioxidants between smokers and non-smokers. Smoking can accelerate the formation of peroxides in vivo, which results in an increase in the level of oxidative stress [34].Previous studies have shown that acetaldehyde, which is a metabolite of ethanol, has cytotoxic, mutagenic, and carcinogenic effects, and can promote the proliferation of cancer cells. Acetaldehyde is also able to combine with DNA, forming stable DNA complexes and generating ROS, resulting in errors in the copying of genetic information and the development of genetic mutations [35–39].

The results of our study suggested that the oxidative stress parameters in patients with CRC are affected by drinking and smoking habits. The proportions of subjects with smoking and drinking habits among the CRC patients and the healthy controls were only a little different, even insignificant, but marginal regarding smoking habit. The serum levels of the oxidative stress parameters were significantly different between the CRC patients and the healthy controls. Regarding whether smoking and drinking habits resulted in differences between the CRC patients and the healthy controls, first, we compared the serum levels of TOS,TAS, and OSI between the healthy controls with (17 peoples) and those without smoking or drinking history(47 peoples). We found significant differences in TOS (p = 0.033) and OSI (p = 0.046), but not in TAS (p = 0.978). Thus, the oxidative stress parameters in the healthy controls were partly affected by drinking and smoking habits. Then, we compared the serum levels of TOS,TAS, and OSI between the CRC patients without smoking and drinking histories (70 patients) and the healthy controls (17 subjects with smoking or drinking history and 47 subjects without smoking or drinking history). The median serum levels of TOS,TAS, and OSI were 20.00μmolH2O2 equiv./L, 1.55 mmol Trolox equiv./L, and 1.27, respectively, in the CRC patients without smoking and drinking histories and 14.20μmolH2O2 equiv./L,1.77 mmol Trolox equiv./L, and 0.80, respectively, in the healthy controls. The differences in the serum levels of TOS,TAS, and OSI between the CRC patients without smoking and drinking histories and the healthy controls were also significant (all p<0.001). Thus, smoking and drinking histories affected the oxidative stress parameters in the patients with CRC and the healthy controls, but the difference in the proportion of subjects with smoking and drinking habits between the CRC patients and the healthy controls did not result in differences in oxidative stress parameters between the two groups.

The present study was designed to compare the levels of oxidative stress parameters between different histopathological grades of CRC. Histological grading of 132 cases of CRC showed that most had intermediate differentiation, whereas approximately 66% of the patients had middle-low grade disease. Among patients with poorly differentiated CRCs, approximately 28% patients underwent colonoscopy sampling, and histological grading could not be performed in patients who had fewer tissue specimens available. Therefore, the purpose of the experimental design could not be fulfilled and follow-up studies are required. Leufkenset al. reported that prediagnostic serum reactive oxygenmetabolite (ROM) levels were associated with increased risk of CRC. However, this association was seen only in subjects with a relatively short follow-up[17]. It was a prospective study and the sample size was large. However, there were some limitations of the study: First, only a single measurement of ROM and FRAP from a baseline blood sample was used and this is not sufficient. Second, the blood samples were collected from 1992 to 1998, but they only tested oxidative stress biomarkers after 15years. It is unclear if a long storage time can affect oxidative stress biomarkers. Third, FRAP method cannot truly reflect the antioxidant levels in a sample. FRAP method practically measures nonprotein total antioxidant capacity, ex. uric acid, bilirubin, vitamin C, Trolox, and polyphenols etc, but the antioxidative effects of proteins cannot be measured or only a small part. It is well known that proteins constitute the main antioxidant component of serum. In addition, the study only detect TAS is not appropriate except the experiment's affecting factors. A our previous study found that independent testing of TAS or TOS might not accurately reflect a subject’s OxS status[40].The reason is, the body’s (or tissue’s or cell’s) oxidation-antioxidant system may remain in a dynamic equilibrium when TAS and TOS simultaneously increases or decreases, so these values alone will not provide oxidative stress. At this point, the OSI can better reflect the oxidative stress status of a subject. Therefore, TAS and TOS should be determined simultaneously, and OSI values should be calculated, could be more beneficial for evaluation of overall oxidative stress status of a subject. It can be seen that our detection index is more reasonable than theirs.

## Conclusion

We showed that serum oxidative stress parameters (TOS, TAS, and OSI) are correlated with the status of CRC, and we suggest that oxidative stress parameters might indicate the severity of CRC; however, further research is required to confirm these findings. Finally, smoking and drinking are important factors that influence oxidative stress parameters, and subsequent research should be conducted excluding these relevant influencing factors.
